# A Comparative Study of the in Vitro Antimicrobial and Synergistic Effect of Essential Oils from *Laurus nobilis* L. and *Prunus armeniaca* L. from Morocco with Antimicrobial Drugs: New Approach for Health Promoting Products

**DOI:** 10.3390/antibiotics9040140

**Published:** 2020-03-25

**Authors:** Ahmed Nafis, Ayoub Kasrati, Chaima Alaoui Jamali, Luísa Custódio, Sara Vitalini, Marcello Iriti, Lahcen Hassani

**Affiliations:** 1 Biology Department, Faculty of Sciences, Chouaïb Doukkali University, Jadida 24000 El, Morocco; ahmed.nafis@edu.uca.ac.ma; 2 Agro-industry department, Private University of Marrakech, Marrakech 42312, Morocco; ayoub.kasrati@gmail.com; 3 Laboratory of Environmental Biology and Sustainable Development, Ecole Normale Superieure, Abdelmalek Essaadi University, Martil 209, Morocco; chaima.tw@gmail.com; 4 Center of Marine Sciences, Faculty of Sciences and Technology, University of Algarve, Ed. 7, Campus of Gambelas, 8005-139 Faro, Portugal; lcustodio@ualg.pt; 5 Department of Agricultural and Environmental Sciences, Milan State University, via G. Celoria 2, 20133 Milan, Italy; sara.vitalini@unimi.it; 6 Laboratory of Microbial Biotechnologies, Agrosciences and Environment, Faculty of Sciences Semlalia, Cadi Ayyad University, Marrakech 40000, Morocco; lhassani@uca.ac.ma

**Keywords:** antimicrobial activity, antibiotic-resistant pathogens, anti-infective agents, pharmacologically active plant-derived natural products, synergistic interaction

## Abstract

*Laurus nobilis* L. (laurel, Lauraceae) and *Prunus armeniaca* L. (apricot, Rosaceae) are important industrial crops and display significant biological properties, including antimicrobial activity. In this work, essential oils (EOs) prepared from the leaves of both species from Morocco were evaluated for the first time for possible synergistic in vitro antibacterial and antifungal effects with some conventional antimicrobial drugs, namely fluconazole, ciprofloxacin and vancomycin. Samples were further evaluated for chemical composition by gas chromatography–mass spectrometry (GC-MS). The main volatile compounds detected in *L. nobilis* were eucalyptol (40.85%), α-terpinyl acetate (12.64%) and methyl eugenol (8.72%), while *P. armeniaca* was dominated essentially by (Z)-phytol (27.18%), pentacosane (15.11%), nonacosane (8.76%) and benzaldehyde (7.25%). Regarding antimicrobial activity, both EOs inhibited significantly all the microorganisms tested. The EO from *L. nobilis* had the highest activity, with minimal inhibitory concentrations (MICs) ranging from 1.39 to 22.2 mg/mL for bacteria and between 2.77 and 5.55 mg/mL for yeasts. Conversely, the combination of the studied EOs with ciprofloxacin, vancomycin and fluconazol resulted in a noteworthy decrease in their individual MICs. In fact, of the 32 interactions tested, 23 (71.87%) demonstrated total synergism and 9 (28.12%) a partial synergistic interaction. The EO from *L. nobilis* exhibited the highest synergistic effect with all the antibiotics used, with fractional inhibitory concentration (FIC) index values in the range of 0.266 to 0.75 for bacteria, and between 0.258 and 0.266 for yeast. The synergistic interaction between the studied EOs and standard antibiotics may constitute promising anti-infective agents useful for treating diseases induced by antibiotic-resistant pathogens.

## 1. Introduction

In recent years there has been increasing interest in the exploitation of natural resources for the identification of new anti-infective agents [1]. Medicinal plants, containing bioactive substances of noteworthy therapeutic value as revealed by their traditional uses and chemical diversity, are considered to be one of the most important sources of such compounds [2,3]. In 2008, more than 80% of the world’s population used medicinal plants and derivatives for their primary care needs [4]. In particular, volatile components such as essential oils (EOs) have attracted much attention because of their benefits for both human health and environmental safety [5].

Nowadays one of the most pressing health problems is the appearance of many types of resistant microbes due to the overuse of antibiotics resulting in a decline in their efficacy [6]. For example, *Staphylococcus aureus* extremely emerged recently and causes potentially fatal nosocomial diseases including necrotizing fasciitis, life-threatening pneumonia and toxinoses like toxic shock syndrome [7]. It is responsible for more than 150,000 affected patients annually in the European Union [8]. Similarly, candidiasis caused by *Candida* species has become a major public health threat, with more than 250,000 affected individuals annually and a mortality rate that exceeds 70% [9]. In fact, the emergence of multidrug-resistant microorganisms is a challenge for the treatment of infections [10], and therefore the development of new approaches to combat these pathogens is sorely needed [11]. Among the strategies under investigation is the combination of antibiotics with non-antibiotic adjuvants, such as EOs, to reduce the minimum effective dose of antimicrobials needed for treatment [10,12].

*Laurus nobilis* L., commonly known as laurel, belongs to the Lauraceae family and is a dioecious evergreen shrub with highly fragrant leaves. Laurel is native to the southern Mediterranean region and is widely cultivated in many regions of the world. Laurel is mainly used in the flavoring industry and also as a botanical biopesticide in postharvest crop protection [13]. Dried laurel leaves are used extensively in cooking, in hair lotions because of their antidandruff activity and for the external treatment of psoriasis [14]. The EO from laurel leaves exhibits several biological properties such as antiviral, antimicrobial and insecticidal activity [13,15,16]. Moreover, it can be used in food preservation [17], cancer treatment [18] and aromatherapy [19].

*Prunus armeniaca* L., known as apricot, is a deciduous fruit tree member of the Rosaceae family. It is a native plant from the East, in particular China and Japan [20], and is considered to be one of the most important temperate fruit trees, with a total production worldwide approaching 2.6 million tons [21]. Apricot has been used in traditional medicine for the treatment of several diseases, for example, its seeds are used to treat hemorrhages, wheeze, asthma, infertility and eye inflammation [22]. Moreover, apricot possess an antimicrobial activity specifically against skin diseases like acne vulgaris, as well as possessing antidandruff activity [23].

In the course of our work to discover new natural anti-infective agents from natural sources, and to boost the valorization of Moroccan plant essential oils, we report here for the first time the synergistic antimicrobial effect of EOs from laurel and apricot leaves with conventional antimicrobial drugs, namely fluconazole, ciprofloxacin and vancomycin. Target microorganisms included mainly those responsible for nosocomial and mycotic diseases. The chemical profiles of the EOs were characterized by gas chromatography–mass spectrometry (GC-MS).

## 2. Results and Discussion

### 2.1. Chemical Composition of the EOs

The preliminary analysis of the GC chromatogram of the studied EOs showed an abundance of several volatile compounds (Table 1). The EO from apricot was characterized by 15 compounds that collectively accounted for 98.33% of the oil’s composition. The EO contained two major compounds, namely (*Z*)-phytol (27.18%) and pentacosane (15.11%). Nonacosane (8.76%), benzaldehyde (7.25%), (*E*)-2-hexenal (6.54%) and heptacosane (6.50%) were also identified in appreciable levels. The EO from laurel was characterized by 14 different compounds representing 98.57% of the total components of the oil. Eucalyptol (40.85%), α-terpinyl acetate (12.64%) and methyl eugenol (8.72%) were the most abundant volatile compounds. The other main components of this EO were linalool (6.81%), α-terpineol (5.60%), eugenol (5.14%) and sabinene (5.13%). Some of these components, for example, (Z)-phytol, eucalyptol, α-terpinyl acetate and linalool, have a number of proven biological activities including antimicrobial and insect-repellant properties [24,25].

The obtained data showed that the composition of the studied laurel EO differed quantitatively to those previously reported in samples from other geographical regions such as Italy, Turkey, Cyprus, Tunisia and Bulgaria. In fact, the concentration of eucalyptol in our samples was lower than the values recorded in Tunisia (56%) [26], Cyprus (58.59%) [27] and Bulgaria (41%) [16]. However, it was higher compared with those found in other studies, namely Algeria (34.62%) [15]. As previously reported, the variability in the chemical composition of the EO can be greatly affected particularly by the geographical location, the climate, the harvest period and genetic factors [6,28].

### 2.2. Antimicrobial and Synergistic Effect of EO

Results of the evaluation of the antimicrobial activity using the disc diffusion method are presented in Table 2 and Table 3. Both EOs exhibited notable potency, with inhibition zones (IZs) ranging from 9.00 mm in the Gram-negative bacteria *Escherichia coli* and *Pseudomonas aeruginosa* (laurel) to 28 mm in the yeast *Candida glabrata* (apricot). The minimum inhibitory concentration (MIC) data obtained by the microdilution method revealed significant activity especially against the *Candida* strains, with values ranging from 2.77 in *C. parapsilosis* to 5.55 mg/mL in the other strains (for laurel) and from 5.84 in *C. glabrata*, *C. krusei* and *C. parapsilosis* to 11.7 mg/mL in *C. albicans* (for apricot). The results showed also that apricot exhibited a moderate efficiency against the Gram-positive bacteria especially towards *S. aureus* and *Bacillus subtilis*, with MIC values of 5.55 and 1.39 mg/mL, respectively. The laurel EO demonstrated the highest activity against all tested Gram-negative bacteria (MIC: 11.7 mg/mL). Concerning the minimal fungicidal concentration (MFC), both EOs exhibited values close to those obtained for MIC with small variations. For example, laurel EO has an effect against *B. subtilis* and *C. parapsilosis* with MFCs of 2.77 and 5.55 mg/mL, respectively. Apricot EO acts against *E. coli* and *Klebsiella pneumoniae* with an MFC of 23.4 mg/mL and *C. parapsilosis* with 11.70 mg/mL. Our findings are in agreement with those reported by several authors for the same species [16,29].

The results of synergistic interaction of both EOs with antibiotics are summarized in Table 4 and Table 5. On the basis of fractional inhibitor concentration index (FICI) values, of 32 interactions tested, 23 (71.87%) represented total synergism and 9 (28.12%) a partial synergistic interaction. The interaction of both EOs with fluconazol resulted in a total synergistic effect against all tested yeasts (FICI from 0.258 to 0.375). For bacterial strains, the interaction of the EO of laurel with the two antibiotics allowed for a total synergism for all bacteria (FICI from 0.266 to 0.5) except for *P. aeruginosa* (FICI: 0.75). The interaction of *P. armeniaca* EO with vancomycin resulted in a partial synergistic effect (FICI: 0.75) and with ciprofloxacin presented a total synergism (FICI from 0.258 to 0.5) except for *B. subtilis* and *M. luteus* (FICI: 0.75).

The gain expressed by the MIC of antibiotics in the presence of EOs at a sub-inhibitory concentration (MIC/4) is summarized in Table 4 and Table 5. Both EOs strongly decreased the antibiotic MICs by 2- to 128-fold against all tested strains. The highly reduced MICs (128-fold) were noted for the combination of the EO from apricot with ciprofloxacin against *P. aeruginosa*, and of laurel with fluconazol against *C. albicans* and *C. glabrata*. The results obtained indicated that the mixture of EOs of both plants with the antimicrobial agents improved the effectiveness of the latter. This improvement can be explained by the presence of high levels of eucalyptol in the EO from laurel, and one major compound in apricot (*Z*-phytol), based on the performed approximate quantification. Eucalyptol is a major component of the EO from several species, in particular the *Eucalyptus* genus [30], and is a recognized antimicrobial agent [31,32]. This compound acts on the plasma membranes and is used for its antitussive effects and in aromatherapy as a skin stimulant [33]. It is also used to treat sinusitis, rheumatism and bronchitis [34]. Phytol exhibits broad-spectrum antimicrobial effects against infective endocarditis bacteria and *Enterococcus faecalis* [31]. Moreover, phytol acts against protein and enzyme inactivation of the important mechanisms of microorganisms [35]. This compound also exhibits several other biological activities such as anti-inflammatory, cytotoxic, metabolic and immune-modulating effects [36,37]. For example, it may prevent types of events such as DNA damage due to its hydroxyl radical scavenging capacity [31]. The synergistic effect demonstrated especially in the case of laurel EO with ciprofloxacin might be due to the correlation between the chemical composition profile and the antimicrobial activity, suggesting that the potential of tested EO could be associated with the highest concentration of eucalyptol known to be an antimicrobial agent that has been previously reported. Plant metabolites that produce a synergistic effect have some generally accepted mechanisms of antimicrobial action including the use of membranotropic agents to enhance antimicrobial diffusion, sequential or dose-dependent effects on common biochemical pathways, and inhibition of protective enzymes [38,39].

## 3. Materials and Methods

### 3.1. Plant Material

Leaves of *L. nobilis* were harvested in February 2019 from Ait Ourir (31°27’N/07°32’W), while *P. armeniaca* leaves were collected in October 2018 from Tassaltante (31°32’N/7°57’W), both in the Marrakech region. The voucher specimens (LANO-41 for *L. nobilis* and PUAR-42 for *P. armeniaca*) were maintained at the Laboratory of Biology and Biotechnology of Microorganisms, Faculty of Sciences Semlalia, Cadi Ayyad University, Marrakech, Morocco. The collected samples were air-dried at room temperature (approximately 25 °C) in the shade for 1 week.

### 3.2. Extraction of EOs

The EOs were extracted from the dried leaves by steam distillation for 4 h using a Clevenger-type apparatus according to Price and Price [40], and yielded (v/w) 2.5% for *L. nobilis* and 1.2% from *P. armeniaca,* based on the dry weight (DW).

### 3.3. Chemical Profiling of the EOs by GC-MS

GC-MS coupled to TG-5MS column (30 m length; 0.25 mm i.d.; 0.25 µm film thickness) and coupled to mass selective detector “ISQ Single Quadrupole Mass spectrometer” (70 eV) was used to characterize both Eos, and the conditions for the analysis were as described previously [28]. Briefly, the injector temperature was 260 °C, helium was the carrier gas, and the temperature program was 1 min at 100 °C ramped from 100 to 260 °C at 4 °C/min and 10 min at 246 °C. Identification of the individual components was assigned by comparing their mass spectra and retention indexes with reference library data [41]. For semi-quantification purposes, the normalized peak area of each compound was used without any correction factors to establish the components’ relative concentrations.

### 3.4. Antimicrobial Evaluation and Determination of Minimum Inhibitory Concentration (MIC) and Minimum Microbicidal Concentration (MMC) 

For the evaluation of the antibacterial activity of the EOs, six pathogenic strains were used, namely *Staphylococcus aureus, Micrococcus luteus, Bacillus subtilis, Escherichia coli*, *Pseudomonas aeruginosa* and *Klebsiella pneumoniae*. In addition, antifungal activity was evaluated using four pathogenic yeasts belonging to the *Candida* genus, namely *C. albicans, C. glabrata, C. krusei* and *C. parapsilosis* (Table 6). All strains were conserved at −80 °C and were grown on Muller Hinton medium for bacteria (24 h) and Sabouraud medium for yeasts (48 h) prior to the assays.

As described by Helal et al. [44] the disc-diffusion method was used to evaluate antimicrobial activity. Petri dishes containing Sabouraud and Muller Hinton (MH) agar were seeded with a cell suspension of yeasts (10^5^ CFU/mL) and bacteria (10^8^ CFU/mL), respectively. Sterile paper discs (6 mm) were individually loaded with 10 µL of each EO and placed on the surface of the previously inoculated media. The plates were incubated at the optimal temperature of each tested microorganism. The antimicrobial potential was evaluated by measuring the inhibition zones (in mm). The microdilution method in 96-well plates was used to determine the MIC which corresponded to the lowest concentration of EO able to inhibit bacterial cell growth [45]. In each microwell, 100 µL of EO dilution (from 93 to 0.36 mg/mL for apricot and 90 to 0.35 mg/mL for laurel) was added to the same volume of yeast inoculum (1–2 10^3^ cells/mL) and bacteria (10^6^ CFU/mL). The last three wells of each row that showed no microbial growth after plate incubation were used to determine the minimum microbicidal concentration (bactericidal and fungicidal). For this, an inoculum from each well was inoculated on Muller Hinton agar plates for bacteria and Sabouraud agar plates for yeasts. The plates were incubated at 37 °C (24 h) and 30 °C (48 h) for bacteria and yeasts, respectively. The MBC and MFC were noted for the concentration where no colonies developed [46]. Fluconazole was used as a positive control for fungi, while ciprofloxacin and vancomycin were used as positive controls for bacteria.

### 3.5. Evaluation of Synergistic Effect of the EOs with Conventional Antibiotics

Three standard antimicrobial agents, namely fluconazol, vancomycin and ciprofloxacin, in combination with each EO were used to evaluate their synergistic effect by the checkerboard assay method described by Nafis et al. [29]. This synergy has been studied by determining the new MIC of antibiotics in the presence of EOs at a lower concentration (MIC/4), based on preliminary tests [47]. Briefly, an aliquot (50 µL) of each EO was added separately to microwells containing 50 µL of different antimicrobial concentrations, and inoculated with 100 µL of cell suspension. As reported by Didry et al. [48], the combined effects were calculated in terms of fractional inhibitor concentration index (FICI), which is most frequently used to define or describe drug interactions and which represents the sum of the FICs of each drug tested. Moreover, the gain was calculated based on the MIC of antibiotic alone and in combination with the EOs.

The FIC index was determined using the following formula: FICI = FIC of EO + FIC of antimicrobial, with FIC of EO = (MIC of EO in combination with antibiotic)/(MIC of EO alone), and FIC of antimicrobial = (MIC of antimicrobial in combination with EO)/(MIC of antimicrobial alone).

The results were interpreted as total synergism when FICI ≤ 0.5, partial synergism when 0.5 < FICI ≤ 0.75, no effect when 0.75 < FICI ≤ 2 or antagonism when FICI > 2.

The MIC gain of the antimicrobials was calculated according to the following formula: MIC gain = MIC of antimicrobial alone/MIC of antimicrobial in combination.

## 4. Conclusions

This work reports the chemical composition and the in vitro antimicrobial activity of EOs from leaves of Moroccan laurel (*L. nobilis)* and apricot (*P. armeniaca),* when applied alone or in combination with conventional antimicrobials, namely fluconazole, ciprofloxacin and vancomycin. The main components of laurel EO were eucalyptol, α-terpinyl acetate and methyl eugenol. The oil from apricot was rich in *Z*-phytol, pentacosane and nonacosane. According to the antimicrobial results, both species displayed notable activity towards all tested strains, and laurel exhibited the highest antimicrobial activity. Additionally, both EOs have a high potential to reduce the individual MICs of all the standard antibiotics tested, especially the EO obtained from laurel. Taken together, our results suggest that EOs from both species should be further investigated as a promising source of natural compounds that can be used in combination with standard antibiotics to combat multidrug-resistant strains.

## Figures and Tables

**Table 1:** Volatile components of essential oils from apricot (*P. armeniaca*) and laurel (*L. nobilis*) leaves. Entries in **bold type** are major components.

RI	Name	Apricot	Laurel
854	(*E*)-2-Hexenal	6.54	-
886	Cyclofenchene	-	2.03
888	*p*-Xylene	3.90	-
954	Ethyltoluene	2.54	-
963	Benzaldehyde	7.25	-
967.2	Sabinen	-	5.13
973	α-Pinene	1.37	2.85
996	Mesitylene	2.62	-
1001	3-Carene	-	1.14
1020	Eucalyptol	-	**40.85**
1030	Limonene	2.54	-
1050	γ-Terpinene	-	1.24
1086	Linalool	6.38	6.81
1164	Terpinen-4-ol	-	4.07
1175	α-Terpineol	-	5.60
1332	α-Terpinyl acetate	-	**12.64**
1339	Eugenol	-	5.14
1370	Methyleugenol	-	**8.72**
1490	β-Cyclogermacrane	-	1.11
1521	γ-Cadinène	1.62	-
1521	Elemicin	-	1.27
1526	δ-Cadinene	0.54	-
1950	(*Z*)-Phytol	**27.18**	-
1969	Hexadecanoic acid	5.48	-
2500	Pentacosane	**15.11**	-
2700	Heptacosane	6.50	-
2900	Nonacosane	**8.76**	-
	Total	98.33	98.57
	Yield	1.20%	2.50%

RI: retention index measured relative to *n*-alkanes (C-9 to C-24) on a non-polar TG-5MS column; -: not detected.

**Table 2:** Inhibition zone diameters, MIC and MBC of essential oils from apricot (*P. armeniaca*) and laurel (*L. nobilis*) leaves, and antibiotics, against bacteria using the disc diffusion and micro-well dilution assays.

Microorganisms	Apricot			Laurel			Ciprofloxacin		Vancomycin	
	IZ	MIC	MBC	IZ	MIC	MBC	IZ	MIC	IZ	MIC
Gram-positive bacteria										
*M. luteus*	12.0 ± 0.10	23.4	23.4	10.0 ± 0.33	22.2	22.20	26.0 ± 0.75	0.01	28.0 ± 0.20	0.001
*S. aureus*	14.0 ± 0.20	23.4	23.4	10.0 ± 0.31	5.55	5.55	27.0 ± 0.40	0.03	27.0 ± 0.32	0.001
*B. subtilis*	14.0 ± 0.05	23.4	23.4	14.0 ± 0.20	1.39	2.77	35.0 ± 1.20	0.01	24.0 ± 0.40	0.125
Gram-negative bacteria										
*E. coli*	18.0 ± 0.25	11.7	23.4	9.00 ± 0.45	>22.5	>22.5	12.0 ± 0.80	0.06	12.0 ± 0.13	0.5
*P. aeruginosa*	22.0 ± 0.12	11.7	11.7	9.00 ± 0.54	22.2	22.2	9.00 ± 0.20	1	11.0 ± 0.24	0.5
*K. pneumoniae*	13.0 ± 0.40	11.7	23.4	9.00 ± 0.60	>22.5	>22.5	8.00 ± 0.82	0.25	11.0 ± 0.19	0.5

IZ: inhibition zone (mm); MIC: minimum inhibitory concentration (mg/mL); MBC: minimum bactericidal concentration (mg/mL).

**Table 3:** Inhibition zone diameters, MIC and MFC of essential oils from apricot (*P. armeniaca*) and laurel (*L. nobilis*) leaves against yeasts using the disc diffusion and micro-well dilution assays.

Microorganisms	Apricot			Laurel			Fluconazol	
	IZ	MIC	MFC	IZ	MIC	MFC	IZ	MIC
**Yeasts**								
*C. albicans*	12.00 ± 0.70	11.70	11.70	10.00 ± 0.21	5.55	5.55	20.00 ± 0.50	1
*C. glabrata*	28.00 ± 0.90	5.85	5.85	13.00 ± 0.12	5.55	5.55	13.00 ± 0.00	1
*C. krusei*	25.00 ± 0.31	5.85	5.85	15.00 ± 0.7	5.55	5.55	24.00 ± 0.80	1
*C. parapsilosis*	26.00 ± 0.81	5.85	11.70	9.00 ± 0.88	2.77	5.55	28.20 ± 0.43	1

IZ: inhibition zone (mm); MIC: minimum inhibitory concentration (mg/mL); MFC: minimum fungicidal concentration (mg/mL).

**Table 4:** Synergistic interaction between laurel (*L. nobilis*) and apricot (*P. armeniaca*) essential oils and ciprofloxacin against resistant bacteria.

Combination	*M. luteus*			*S. aureus*			*B. subtilis*			*E. coli*			*P. aeruginosa*			*K. pneumoniae*		
	FIC	FICI	Gain	FIC	FICI	Gain	FIC	FICI	Gain	FIC	FICI	Gain	FIC	FICI	Gain	FIC	FICI	Gain
*P. armeniaca* EO	0.25	-	-	0.25	-	-	0.25	-	-	0.25	-	-	0.25	-	-	0.25	-	-
Ciprofloxacin	0.5	0.75^b^	2	0.25	0.5^a^	4	0.5	0.75^b^	2	0.062	0.312^a^	16	0.008	0.258^a^	128	0.125	0.375^a^	8
Vancomycin	0.5	0.75^b^	2	0.5	0.75^b^	2	0.5	0.75^b^	2	0.25	0.75^b^	4	0.5	0.75^b^	2	0.5	0.75^b^	2
*L. nobilis* EO	0.25	-	-	0.25	-	-	0.25	-	-	0.25	-	-	0.25	-	-	0.25	-	-
Ciprofloxacin	0.25	0.5^a^	4	0.062	0.312^a^	16	0.25	0.5^a^	4	0.125	0.375^a^	8	0.016	0.266^a^	64	0.063	0.313^a^	16
Vancomycin	0.247	0.497^a^	4	0.247	0.497^a^	4	0.125	0.375^a^	8	0.25	0.5^a^	4	0.5	0.75^b^	2	0.125	0.375^a^	8

FIC: fractional inhibitory concentration; FICI: fractional inhibitor concentration index; FIC of oil = (MIC of EO in combination with antibiotic)/(MIC of EO alone); FIC of antibiotic = (MIC of antibiotic in combination with EO)/(MIC of antibiotic alone); FIC index = FIC of EO + FIC of antibiotic. ^a^ Total synergism; ^b^ Partial synergism.

**Table 5:** Synergistic interaction between laurel (*L. nobilis*) and apricot (*P. armeniaca*) essential oils and fluconazol against clinical pathogenic yeasts.

Combination	*C. albicans*			*C. glabrata*			*C. krusei*			*C. parapsilosis*		
	FIC	FICI	Gain	FIC	FICI	Gain	FIC	FICI	Gain	FIC	FICI	Gain
*P. armeniaca* EO	0.25	-	-	0.25	-	-	0.25	-	-	0.25	-	-
Fluconazole	0.031	0.281^a^	32	0.016	0.266^a^	64	0.125	0.375^a^	8	0.063	0.313^a^	16
*L. nobilis* EO	0.25	-	-	0.25	-	-	0.25	-	-	0.25	-	-
Fluconazole	0.008	0.258^a^	128	0.008	0.258^a^	128	0.16	0.266^a^	64	0.16	0.266^a^	64

FIC: fractional inhibitory concentration; FICI: fractional inhibitor concentration index; FIC of oil = (MIC of EO in combination with antibiotic)/(MIC of EO alone); FIC of antibiotic = (MIC of antibiotic in combination with EO)/(MIC of antibiotic alone); FIC index = FIC of EO + FIC of antibiotic. ^a^ Total synergism.

**Table 6:** Bacterial and yeast strains used in this study.

Bacteria	Access number	Reference
*Staphylococcus aureus*	CCMM B3	[42]
*Micrococcus luteus*	ATCC 10240	[42]
*Bacillus subtilis*	ATCC 9524	[42]
*Escherichia coli*	ATCC 8739	[42]
*Pseudomonas aeruginosa*	ATCC 10240	[42]
*Klebsiella pneumoniae*	Clinically isolated	[42]
Yeasts		
*Candida albicans*	CCMM-L4	[43]
*Candida glabrata*	CCMM-L7	[43]
*Candida krusei*	CCMM-L10	[43]
*Candida parapsilosis*	CCMM-L18	[43]
